# Attenuation of Salt-Induced Cardiac Remodeling and Diastolic Dysfunction by the GPER Agonist G-1 in Female mRen2.Lewis Rats

**DOI:** 10.1371/journal.pone.0015433

**Published:** 2010-11-03

**Authors:** Jewell A. Jessup, Sarah H. Lindsey, Hao Wang, Mark C. Chappell, Leanne Groban

**Affiliations:** 1 The Department of Physiology and Pharmacology, Wake Forest University School of Medicine, Winston Salem, North Carolina, United States of America; 2 The Hypertension and Vascular Research Center, Wake Forest University School of Medicine, Winston Salem, North Carolina, United States of America; 3 The Department of Anesthesiology, Wake Forest University School of Medicine, Winston Salem, North Carolina, United States of America; Istituto Dermopatico dell'Immacolata, Italy

## Abstract

**Introduction:**

The G protein-coupled estrogen receptor (GPER) is expressed in various tissues including the heart. Since the mRen2.Lewis strain exhibits salt-dependent hypertension and early diastolic dysfunction, we assessed the effects of the GPER agonist (G-1, 40 nmol/kg/hr for 14 days) or vehicle (VEH, DMSO/EtOH) on cardiac function and structure.

**Methods:**

Intact female mRen2.Lewis rats were fed a normal salt (0.5% sodium; NS) diet or a high salt (4% sodium; HS) diet for 10 weeks beginning at 5 weeks of age.

**Results:**

Prolonged intake of HS in mRen2.Lewis females resulted in significantly increased blood pressure, mildly reduced systolic function, and left ventricular (LV) diastolic compliance (as signified by a reduced E deceleration time and E deceleration slope), increased relative wall thickness, myocyte size, and mid-myocardial interstitial and perivascular fibrosis. G-1 administration attenuated wall thickness and myocyte hypertrophy, with nominal effects on blood pressure, LV systolic function, LV compliance and cardiac fibrosis in the HS group. G-1 treatment significantly increased LV lusitropy [early mitral annular descent (e′)] independent of prevailing salt, and improved the e′/a′ ratio in HS versus NS rats (P<0.05) as determined by tissue Doppler.

**Conclusion:**

Activation of GPER improved myocardial relaxation in the hypertensive female mRen2.Lewis rat and reduced cardiac myocyte hypertrophy and wall thickness in those rats fed a high salt diet. Moreover, these advantageous effects of the GPER agonist on ventricular lusitropy and remodeling do not appear to be associated with overt changes in blood pressure.

## Introduction

Hypertension in postmenopausal women results in left ventricular hypertrophy (LVH), a major causative factor for reductions in both myocardial relaxation and diastolic compliance which are key components of diastolic dysfunction [1]–[3]. In addition to the loss of estrogen, salt sensitivity of blood pressure in premenopausal normotensive and hypertensive women has been attributed to an increased risk of subsequent age-related hypertension [4]. Salt-sensitive hypertensive patients have a higher incidence of LVH than salt-resistant hypertensive individuals [5], further increasing their predisposition for the development of diastolic heart disease. Indeed, the evolution of LVH differs between pre-and postmenopausal women, with a greater induction of hypertrophy when circulating estrogen levels are reduced [6]. While evidence suggests that estrogen may protect the premenopausal heart from the deleterious effects of hypertension and salt-induced LV remodeling [7], [8], the mechanisms and receptors involved remain unclear.

Estrogen attenuates the development of cardiac hypertrophy and fibrosis through activation of steroid receptors ERα and ERβ located on myocytes, fibroblasts, and the extracellular matrix [9]. Estrogen also interacts with the novel G protein-coupled receptor GPR30 or GPER, located at the cell membrane and endoplasmic reticulum [10], [11]. Signaling pathways for GPER include mitogen-activated protein kinase (MAPK) and phosphoinositide 3-kinase (PI3K), which can modulate nuclear transcriptional events similar to the classic ERs [12]. GPER binds estradiol at a similar affinity as ERα and ERβ and exerts comparable actions on calcium mobilization and PI3K activation [13]. GPER activation improves contractile function and reduces infarct size in isolated rat and mouse hearts subjected to ischemia/reperfusion injury [14]; however, the precise role for GPER in cardiac remodeling is not known.

Our recent studies find that chronic treatment with the selective GPER agonist G-1 significantly reduces hypertension in the estrogen depleted mRen2.Lewis congenic rat [15] but fails to attenuate the elevated systolic blood pressure characteristic of estrogen-intact littermates. In addition to estrogen-sensitivity, mRen2.Lewis females also exhibit salt-sensitivity whereby high sodium intake in estrogen-replete mRen2.Lewis increases blood pressure, initiates LV hypertrophy, and impairs diastolic function [16]–[19]. As our preliminary data indicate that high salt increased GPER gene expression within the heart, we hypothesized that chronic GPER activation attenuated changes in LV remodeling due to prolonged intake of a high salt diet.

## Materials and Methods

### Animals

Female mRen2.Lewis rats were obtained from the Hypertension and Vascular Research Center Congenic Colony of Wake Forest University School of Medicine and all studies were approved by the institution's Animal Care and Use Committee under protocol #A08-198. Rats were individually housed in an Association for Assessment and Accreditation of Laboratory Animal Care-approved, temperature (22±2°C) and light (12 h light/dark cycle) controlled facility with *ad libitum* access to rat chow and tap water.

### Experimental protocol

The mRen2.Lewis rats were fed either a normal salt (0.5% sodium; NS, n = 12) or high salt (4% sodium; HS, n = 10) diet (Harlan TEKLAB, Madison, WI) for 10 weeks starting at 5 weeks of age. The percentage of sodium was chosen based upon its known effects on cardiovascular pathogenesis in the mRen2.Lewis female rat, as originally determined [17]–[19]. At 13 weeks of age, when salt-induced blood pressure elevations plateau [18], animals received either the selective GPER agonist G-1 (NS, n = 5; HS, n = 5) or vehicle (DMSO/EtOH; NS, n = 7; HS, n = 5) administered via osmotic minipump at 40 nmol/kg/hr for 2 weeks (Alzet Model 2ML2, Durect Corporation, Cupertino, CA). The dose of G-1 used was based upon the initial study conducted in our laboratory that demonstrated significant blood pressure reductions over a two week period using 40 nmol/kg/hr in estrogen-deplete mRen2.Lewis female rats [15]. Systolic blood pressure was measured throughout the study using tail-cuff plethysmography (Narco Bio-systems, Houston, TX) as previously described and validated versus telemetry [20].

The pressures were recorded in pre-trained rats while lightly restrained and warmed (35°C). At the end of the 2-week treatment period, left ventricular dimensions and left ventricular function were assessed by M-mode and Doppler echocardiography, respectively. Once the experiments were terminated, the hearts were removed and weighed with a portion being submerged in 10% formalin for histology.

### Echocardiographic studies

Echocardiography of all animals was performed at the end of week 15 using a Philips 5500 or Envisor CD echocardiograph (Philips Medical Systems, Andover, MA) and a 12 MHz phased array probe. All measurements were made in accordance with the conventions of the American Society of Echocardiography, and were conducted by the same investigator who was blinded to the experimental groups. For the procedure, animals were lightly sedated with 4% isoflurane, and then maintained at 2.0% isoflurane. Left ventricular end-diastolic and end-systolic diameters (LVEDD and LVESD, respectively), LV posterior wall thickness (PWT), and anterior wall thickness (AWT) were measured from midpapillary, short-axis images obtained by M-mode echocardiography. The percentage of LV fractional shortening (%FS), an index of contractile function, was calculated as FS (%)  =  [(LVEDD – LVESD)/LVEDD]×100. LV mass was calculated using a standard cube formula, which assumes a spherical LV geometry according to the formula: LV mass (LVmass)  = 1.04×[[LVEDD + PWT + AWT]^3^ – LVEDD], where 1.04 is the specific gravity of muscle. Relative wall thickness (RWT) was calculated as: 2× PWT/LVEDD. Mitral inflow measurements of early filling velocity (E_max_), deceleration slope of early filling velocity (E_dec_ slope), and deceleration time of early filling flow velocity (E_dec_ time) were obtained using pulsed Doppler, with the sample volume placed at the tips of mitral leaflets from an apical four-chamber orientation. The following measurements were made from the septal mitral annular velocity by tissue Doppler imaging (DTI): early diastolic (e′), late diastolic (a′), and the ratio, e′/a′. The early filling velocity-to-early mitral annular velocity ratio (E/e′) was used as a measure of filling pressure. All measurements were performed with an off-line analysis system (Xcelera 3.1, Koninklijke Philips Electronics, Netherlands) by the masked investigator. An average of at least five consecutive cardiac cycles to minimize beat-to-beat variability was used for all measured and calculated systolic and diastolic indices.

### Histopathology

Heart specimens were fixed for 24 h in 10% formalin, then dehydrated in graded ethanols and embedded into paraffin blocks. 4 µm sections were mounted on positive-charged slides and stained with Picrosirius red (PSR) to evaluate collagen deposition within the tissue. Images of interstitial and perivascular collagen were captured using both bright-field and polarized dark-field microscopy in order to clearly differentiate the Sirius red bound collagen from other cellular structures [21], [22]. The ratios of collagen positive stained pixels to unstained pixels were calculated using Adobe Photoshop Creative Suite 3. Myocyte cross-sectional area was measured from slides stained with hematoxylin and eosin. One hundred cardiomyocytes from each section (25 from each of LV anterior wall, posterior wall, free wall and septum) were analyzed at 400× magnification using Simple PCI 6.0 software connected to a Leica DM4000B (Bannockburn, IL) and an Olympus polarizing microscope system (Center Valley, PA), respectively. Bright field photomicrographs were captured with a Leica DFC digital camera and processed using Leica Application Suite software, while polarized images were taken using Diagnostic Instruments Inc. Digital SPOT RT, 3-pass capture, thermoelectrically cooled charge-coupled camera (Sterling Heights, MI) and processed using the SPOT® Advanced software. A blinded observer took 2 images from each of 4 randomized quadrant fields totaling 8 images per section under bright field and with polarization magnified 200-times. The digitized images of equal pixel composition were analyzed using Adobe Photoshop Creative Suite 3. The quantified collagen content was determined as a ratio of PSR-stained pixels divided by total pixels.

### Immunohistochemistry

Heart sections mounted on slides were blocked with 0.1% Tween, 1% bovine serum albumin, and 5% normal donkey serum. Anti-GPER (MBL, Woburn, MA) was diluted 1∶250 in blocking buffer and incubated overnight at 4°C. For a negative control, the primary antibody was pre-incubated with the blocking peptide for 1 h at 25°C and centrifuged before being applied to tissue sections. Biotinylated goat anti-rabbit (VectorLabs, Burlingame, CA) was diluted 1∶400 in the blocking buffer and applied for 1 h at 25°C. Antibody binding was detected using Vectastain Elite avidin-biotin complex kit (VectorLabs) and 0.1% diaminobenzene (Sigma, St. Louis, MO). Slides were counterstained with hematoxylin (Sigma, St. Louis, MO).

### Analysis of gene expression by quantitative real-time PCR

Real-time PCR was used to detect cardiac mRNA expression levels for GPER and brain natriuretic peptide (BNP). Total RNA was extracted from frozen, pulverized left ventricular tissue from each rat using TRIzol Reagent (Invitrogen, Carlsbad, CA, USA) and processed according to the recommendations of the manufacturer. The quality and quantity of RNA samples were determined by spectrometry and agarose gel electrophoresis. Complimentary first strand DNA was synthesized from oligo (dT) primed total RNA using the Omniscript RT kit (Quiagen Inc, CA, USA). Relative quantification by real-time PCR was performed using SYBR Green PCR kit (Quiagen, CA, USA). Amplification and detection were performed with the use of the ABI7500 Sequence Detection System (Applied Biosystems). PCRs were carried out in duplicate. Only one peak from the dissociation curve was found from each pair of oligonucleotide primers tested. In each PCR run, a no-template-control was included to check for any contamination disturbing PCR. It was also confirmed that no amplification occurred when samples were not subjected to reverse transcription. Sequence-specific oligonucleotide primers were designed according to published GenBank (www.ncbi.nlm.nih.gov/Genbank) sequences and confirmed with OligoAnalyzer 3.0 (Table 1). The relative target mRNA levels in each sample were normalized to S16 ribosomal RNA. Expression levels are given relative to the geometric mean of the control group.

**Table 1:** Sequence of PCR Primers.

Gene	Oligonucleotide Sequence	GenBank Locus	Position	Product size (bp)
S16	GGAGAGATTTGCTGGTGTGG TCCGATCGTACTGGATGAGG	BC159438.1	223-242395-376	120
GPER	TCTACCTAGGTCCCGTGTGG AGGCAGGAGAGGAAGAGAGC	NM_133573.1	86-105236-217	151
BNP	GCCAGTCTCCAGAACAATCC CCTTGGTCCTTTGAGAGCTG	NM_031545.1	155-174251-232	97

S16; GPER, G protein-coupled estrogen receptor; BNP, brain natriuretic peptide.

### Statistical analysis

All values are expressed as mean ± SEM. Blood pressures were analyzed by two-way ANOVA, followed by the Bonferroni post-*hoc* analysis. Data from physical characteristics, echocardiography, and histopathology were compared using two-way ANOVA to determine significant effects of either salt or G-1. If the interaction was significant, between group comparisons were conducted by using the Bonferroni post-*hoc* test. Data from real-time PCR was analyzed by performing one-way ANOVA followed by Tukey's post-*hoc* test. All data were analyzed using GraphPad PRISM Version 5 (GraphPad, San Diego, CA, USA) with P<0.05 considered statistically significant.

## Results

**Figure 1:**
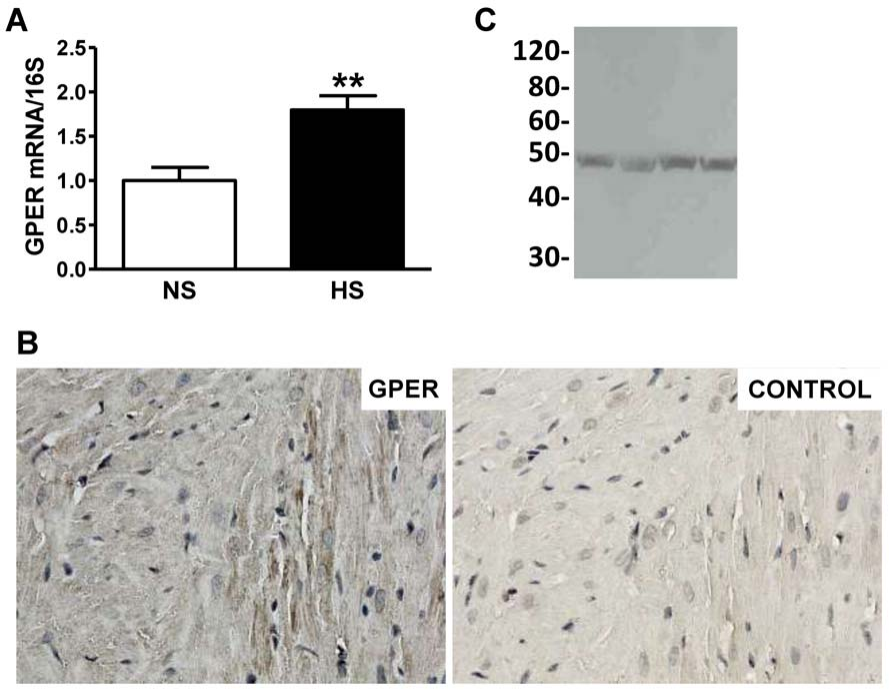
Cardiac GPER expression increases in salt-fed mRen2.Lewis females. **a.** Cardiac GPER gene expression in female mRen2.Lewis rats fed a normal salt (NS) or high salt (HS) diet determined by real-time PCR and expressed as the ratio of GPER mRNA to S16 ribosomal RNA. Values are mean ± SEM; ** P<0.01. **b.** Left, representative GPER immunostaining in a cross section of the heart from an intact mRen2.Lewis female. Right, representative control section stained with pre-adsorbed primary antibody. **c.** Full-length immunoblot of cardiac membranes showing the specificity of the primary antibody using for immunohistochemistry as evidenced by a single band corresponding to the appropriate molecular weight of GPER (∼50 kDa).

As previously reported [18], the chronic high salt diet significantly increased systolic blood pressure in the female mRen2.Lewis by nine weeks of age (215±19 vs. 147±27 mmHg; P<0.001). To determine whether cardiac GPER gene expression was altered by high salt, we utilized real-time PCR to compare GPER mRNA in hearts from female mRen2.Lewis rats fed a normal salt (NS) or high salt (HS) diet. GPER mRNA was increased by 80% in HS versus NS hearts (Figure 1A), prompting further investigation of the role of GPER in salt-induced cardiac remodeling and diastolic dysfunction. Immunohistochemistry of fixed heart sections using an antibody directed against GPER showed prominent staining in cardiomyocytes (Figure 1B). Staining was attenuated by pre-incubation of the primary antibody with the immunizing peptide. As shown in Figure 1C, analysis of cardiac homogenates by Western blot using the same antibody revealed a single immunoreactive band at ∼48 kDa, similar to that reported in aortic tissues [15]. This molecular weight of GPER is slightly higher than the predicted molecular weight of 38 kDa and is likely due to post-translational glycosylation [10].

**Figure 2:**
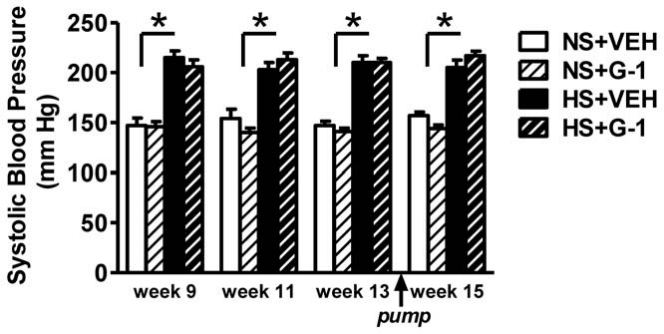
G1 treatment for two weeks does not affect salt-associated increases in blood pressure. Tail-cuff systolic blood pressure in conscious female mRen2.Lewis rats fed a normal salt (NS) or high salt (HS) diet over the course of the experiment. Arrow indicates time of pump insertion for 14-day delivery of either G-1 or vehicle (VEH). Values are mean ± SEM; * P<0.05 compared to NS (Salt effect).

Two-weeks of the GPER agonist G-1 infusion in either the HS or the NS group did not significantly alter systolic blood pressure [G-1: F(1,18) = 3.88, P = 0.064), despite the fact that the HS diet markedly exacerbated systolic hypertension in comparison to NS (Figure 2). The lack of a blood pressure response to G-1 in the intact mRen2.Lewis females fed a NS diet is consistent with previous results in the female mRen2.Lewis strain [15].

**Figure 3:**
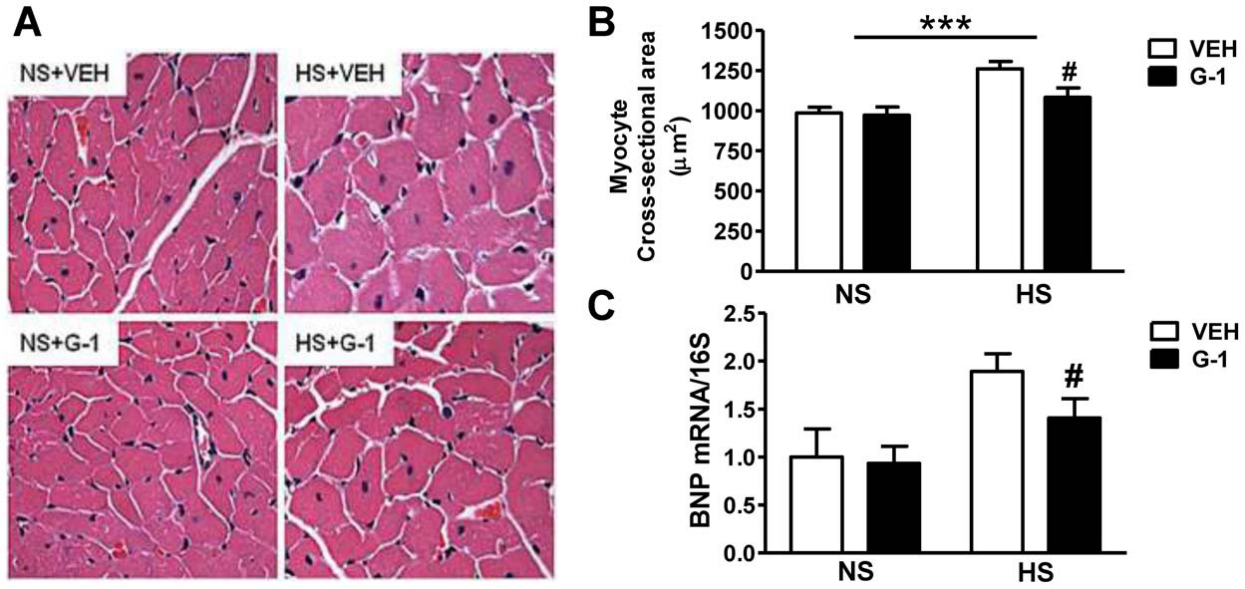
GPER activation with G1 limits salt-induced myocyte hypertrophy. **a.** Representative hematoxylin and eosin staining outlining cardiomyocytes. **b.** Quantification of myocyte cross-sectional area. Data represent mean ± SEM. *** P<0.001 (Salt effect); # P<0.05 (G-1 effect). **c.** Cardiac BNP gene expression determined by real-time PCR and expressed as the ratio of BNP mRNA to S16 ribosomal RNA. # P<0.05 (G-1 effect).

The effects of HS on cardiac hypertrophy and body weight change, in the presence or absence of G-1, are shown in Table 2. The body weight of HS females (203±7 grams) was not significantly different from NS rats (216±4 grams) after 8-weeks of the dietary intervention. The weight gain that occurred over the 2-week infusion period was independent of treatment (G-1: F(1,18) = 0.22, P = 0.64). As expected, heart weight normalized to body weight was greatest in HS rats. Although GPER activation did not influence cardiac mass in NS females, salt-induced cardiac hypertrophy was attenuated by G-1 (Salt × G-1: F(1,18) = 7.404, P<0.01). Microscopic examination of ventricular cross-sections revealed a 27% increase in overall myocyte cross-sectional area (P<0.001) in HS rats compared to NS littermates, and this salt-induced effect was attenuated by G-1 administration (P<0.05) (Figures 3A and 3B). These observations were further supported by a salt-induced increase in BNP gene expression, a biomarker of cardiomyocyte hypertrophy. HS increased cardiac BNP mRNA by two-fold, and G-1 treatment attenuated this effect (Figure 3C).

**Table 2:** Physical Characteristics of the Four Experimental Groups.

			P-value		
	Normal Salt	High Salt	Salt	G-1	Interaction
**Body weight gain, %**					
VehicleG-1	2.16±0.82.24±0.8	3.00±0.83.75±0.9	0.20	0.64	0.71
**Whole heart weight, g**					
VehicleG-1	0.71±0.010.75±0.02	1.09±0.051.11±0.05	**<0.01**	0.39	0.77
**Heart/body weight, mg/g**					
VehicleG-1	3.28±0.073.37±0.09	5.39±0.084.98±0.13	**<0.01**	0.10	**0.01**

Data are expressed as mean ± SEM. Body weight gain represents the percent change in weight from week 13 (prior to pump insertion) to week 15.

**Figure 4:**
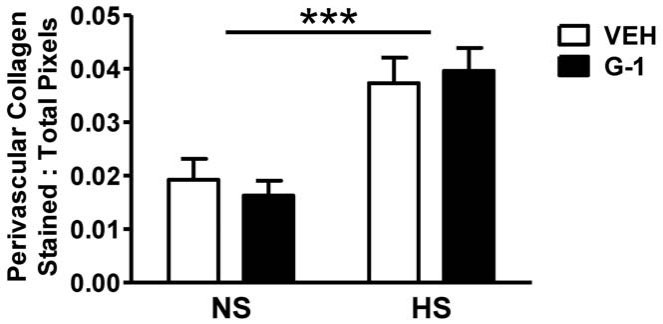
G1 treatment for two weeks does not alter salt-induced increases in collagen deposition. Quantification of cardiac perivascular collagen content using polarized dark field images. Data represent mean ± SEM. *** P<0.001 (Salt effect).

Long-term HS significantly increased interstitial and perivascular collagen deposition as measured by Picrosirius Red staining (Figure 4). The increase in cardiac fibrosis resulting from the HS diet was not affected by G-1 administration (P>0.05). Cardiac collagen gene expression was also not altered by G-1 (data not shown). G-1 did not influence the deposition of cardiac collagen in NS animals.

Left ventricular wall dimensions and systolic parameters are summarized in Table 3. M-mode examination revealed that LVESD was significantly increased by HS and was associated with reductions in percent fractional shortening. As expected, relative wall thickness was greater in HS rats when compared to NS littermates. While salt contributed to 30% of the variance in wall thickness, G-1 also had a specific effect, accounting for 15% of the total variance in RWT. Importantly, G-1 treatment decreased wall thickness, despite minimal differences in blood pressure between salt-fed groups.

**Table 3:** Cardiac Geometry and Systolic Parameters.

			P-value		
	Normal Salt	High Salt	Salt	G-1	Interaction
**LV mass**					
VehicleG-1	3.67±0.352.85±0.28	7.55±0.467.56±0.79	**<0.001**	0.423	0.410
**LVEDd, cm**					
VehicleG-1	0.66±0.020.68±0.02	0.66±0.020.73±0.02	0.174	**0.045**	0.213
**LVESd, cm**					
VehicleG-1	0.35±0.010.31±0.03	0.43±0.010.46±0.02	**<0.001**	0.930	0.066
**RWT**					
VehicleG-1	0.52±0.050.46±0.05	0.70±0.030.56±0.03	**<0.001**	**0.042**	0.388
**%FS**					
VehicleG-1	46±154±3	35±237±2	**<0.001**	0.053	0.181

Data are expressed as mean ± SEM. LV mass  =  left ventricular mass; LVEDd  =  left ventricular end-diastolic dimension; LVESd  =  left ventricular end-systolic dimension; RWT  =  relative wall thickness; %FS  =  percent fractional shortening.

**Figure 5:**
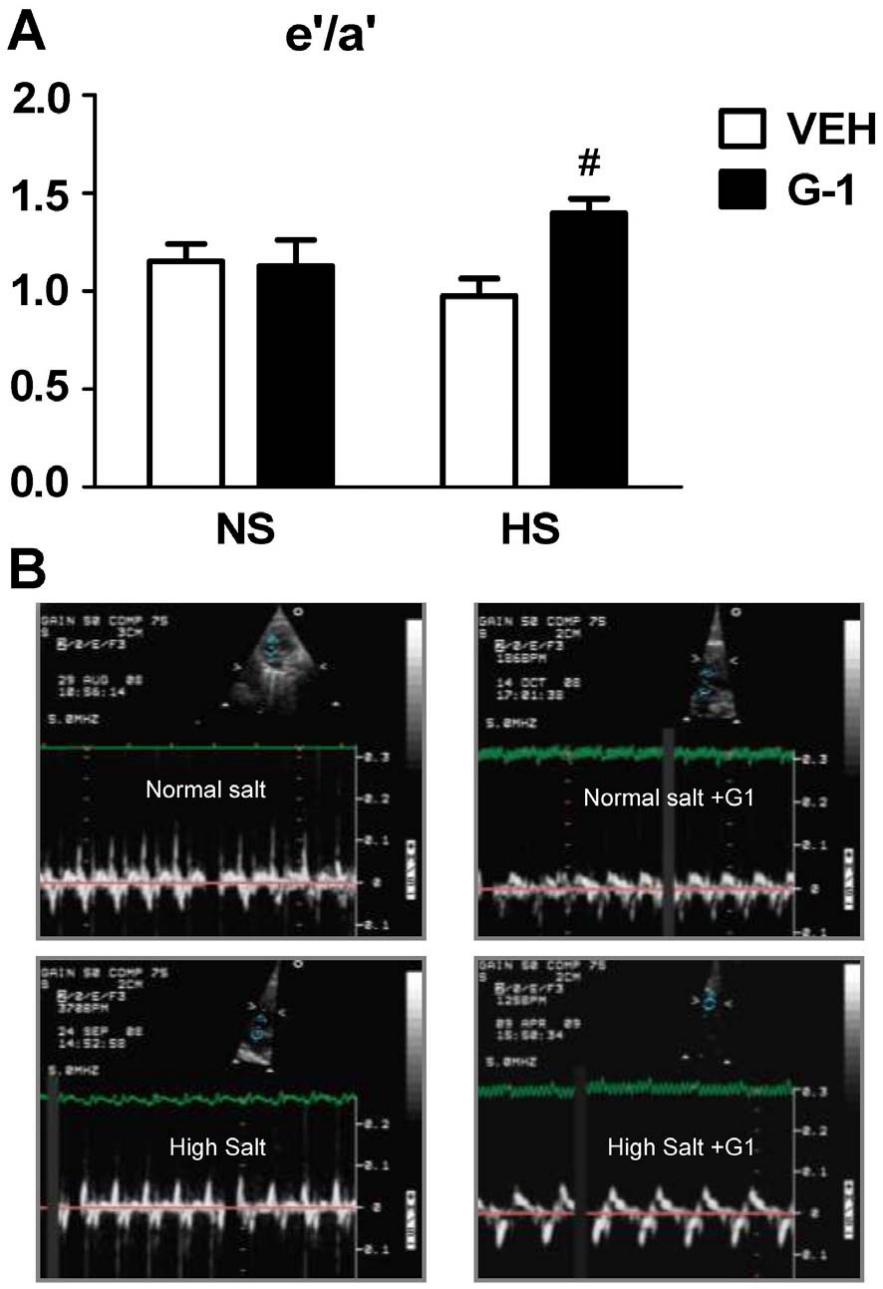
GPER activation with G1 improves myocardial relaxation in normal- and salt-fed mRen2.Lewis females. **a.** Data represent mean ± SEM. e'/a′  =  early mitral annular velocity-to-late mitral annular velocity ratio. # P<0.05 compared to NS +G-1 (Salt x G-1 interaction: P = 0.036). **b.** Representative tissue Doppler images of early (e') and late (a') septal mitral annular velocities from each treatment group.

In addition to salt-associated changes in cardiac geometry, diastolic parameters were also negatively affected by the HS diet (Table 4). Conventional Doppler showed evidence of reduced ventricular compliance in HS rats as manifested by a shorter deceleration time of the E wave and an increased early deceleration slope as compared to NS rats. G-1 administration minimally affected these early mitral inflow parameters, likely due to a G-1 × salt interaction on heart rate (G-1 × Salt: F(1,18) = 6.70, P = 0.019). Indeed, conventional transmitral Doppler measures of diastolic function are load and heart rate dependent [23]–[26]. Reductions in preload can lead to reductions in early transmitral filling velocity and in the ratio of early-to-late LV filling (E/A). Conversely, increases in preload entail a transition towards a pseudonormal pattern (e.g., E>A) or reduced compliance. Heart rate and rhythm are other factors that affect LV diastolic filling, independent of diastolic function. For instance, at higher heart rates (shorter diastolic filling time), the late mitral inflow velocity (A), may be increased as it becomes superimposed on the E deceleration slope. When the diastolic interval is very short (e.g., rat heart rates >300 beats/min), there could be fusion of the E and A velocities. The inclusion of tissue Doppler, however, facilitates the assessment of diastolic function since it is less affected by loading conditions than conventional Doppler [27], and the tissue Doppler surrogate of LV filling pressure, E/e′, has been shown to be relatively independent of heart rate and rhythm abnormalities [28]–[30]. In the present study, diastolic function as assessed by tissue Doppler showed that G-1 administration significantly improved myocardial relaxation in both diet regimens, as manifested by an increase in mitral annular descent (e′) (Table 3). GPER activation, irrespective of salt, accounted for 41% of the variance in e′, whereas salt alone contributed to just 10% of the variance in this measure of ventricular lusitropy. Another manifestation of myocardial relaxation, the ratio of early to late mitral annular velocity, was increased with G-1 treatment in the HS group while the GPER agonist had minimal effects in NS-fed rats (Figure 5). Specifically, the combination of G-1 and HS had a synergistic effect on e′/a′, accounting for 20% of the variance of this tissue Doppler measure of diastolic function.

**Table 4:** Heart Rate and Diastolic Parameters.

			P-value		
	Normal Salt	High Salt	Salt	G-1	Interaction
**Heart rate, bpm**					
VehicleG-1	374±5392±3	422±9403±10	**<0.001**	0.945	**0.019**
**Emax, cm/s**					
VehicleG-1	66±471±2	67±372±3	0.832	0.153	0.948
**E dec time, sec**					
VehicleG-1	0.053±0.0030.046±0.004	0.033±0.0020.032±0.002	**<0.001**	0.199	0.218
**E dec slope, cm/s^2^**					
VehicleG-1	12±116±2	21±223±1	**<0.001**	0.084	0.734
**e', cm/s**					
VehicleG-1	5.6±0.26.8±0.3	5.1±0.46.2±0.3	0.087	**0.002**	0.846
**E/e'**					
VehicleG-1	11.7±0.510.6±0.3	13.5±1.511.8±0.8	0.115	0.12	0.753

Data are expressed as mean ± SEM. Emax  =  maximum early transmitral filling velocity; E dec time  =  early-filling deceleration time; E dec slope  =  early-filling deceleration slope; e' =  early mitral annular velocity; E/e'  =  early transmitral filling velocity-to-mitral annular velocity ratio.

## Discussion

To our knowledge, this is the first study investigating the cardiac structural and functional effects of *in vivo* activation of the novel receptor GPER in the female mRen2.Lewis rat, a congenic rodent model of salt-sensitive hypertension and early diastolic dysfunction [17]–[19]. The most significant effects of G-1 treatment were attenuation of salt-induced LV wall thickness, myocyte hypertrophy, and early diastolic function via mechanisms that are independent of systemic blood pressure. We also found that GPER activation improved LV lusitropy in both the presence and absence of high salt. Given that G-1 is not a ligand for estrogen receptors α or β [31] and that GPER immunostaining was present on cardiomyocytes, these findings suggest important anti-remodeling and lusitropic roles for GPER that likely reflect direct actions on cardiac tissue. Indeed, endogenous estrogen could contribute to GPER-mediated cardioprotection, since estradiol binds GPER with an affinity similar to G-1 [31]. The focus on diastolic dysfunction pathogenesis and cardiac remodeling is of particular importance in the aging female population given the clinical evidence of a preponderance of heart failure with normal ejection fraction [2], [32], [33]. In addition, there are no established therapies to optimally treat this form of diastolic heart disease or prevent its occurrence [34].

Chronic high salt intake stimulates structural changes in the left ventricle through pressure overload and via direct myocardial effects [35]. LV remodeling is characterized by an increase in LV mass and fibrosis, with the myocyte and fibroblast as the predominant cell types involved [36]. The mRen2.Lewis female rat exhibited salt-dependent increases in blood pressure and cardiac hypertrophy with concomitant perivascular collagen deposition, consistent with our previous work in this strain [18], [19]. While the presence of endogenous estrogen in the intact mRen2.Lewis female affords some cardioprotection in the face of high salt [19], cardiac GPER gene expression was significantly increased by HS, indicating a role for this receptor in salt-induced cardiac remodeling. The additive benefit of G-1 administration late in the remodeling process and the localization of GPER on cardiac myocytes suggest cardioprotective actions independent of ERα or ERβ [37].

Estrogen retards the progression of hypertension [6], delays or attenuates the development of cardiac hypertrophy [2], [38], limits fibrosis, and reduces the activation of the renin-angiotensin system [39]–[41] in salt-sensitive experimental models and in humans. Estrogen attenuates myocyte growth and LVH by blocking MAPK activity [42] and various protrophic components of the cardiac renin-angiotensin system [43]–[45]. The present data demonstrate a protective effect of GPER activation on salt-induced myocyte growth and myocardial hypertrophy, as defined by heart weight-to-body weight ratio and relative wall thickness. These protective effects of G-1 were pressure-independent and were accompanied by attenuation of the salt-induced stimulation of cardiac BNP gene expression. Cardiac BNP has been utilized as a biomarker of cardiomyocyte hypertrophy and cardiac dysfunction [46], [47].

Estrogen's anti-remodeling effects also involve its actions on fibroblasts and the cardiac extracellular matrix, composed primarily of collagen proteins. A shift in fibrillar collagen from type III (“youthful” collagen) to type I, for example, is the primary determinant of LV diastolic stiffness [48]. In aged [49] and salt-sensitive rats [50], ovariectomy increases collagen I and reduces collagen III and the metalloproteinase that degrades collagen, MMP2, while estrogen replacement reverses these effects [49]. Likewise, we previously showed limited interstitial cardiac collagen deposition in the estrogen-intact HS mRen2.Lewis when compared to ovariectomized HS and NS littermates [19]. In the present study, G-1 did not alter perivascular collagen deposition; however, the lack of a potent G-1-mediated anti-fibrotic effect under high salt conditions may be obscured by the presence of endogenous estrogen. In addition, if the extent of cardiac collagen is a reflection of exacerbated hypertension, we would expect this fibrosis to persist given that G-1 had minimal effects on blood pressure. On the contrary, the absence of a G-1 effect on collagen deposition may imply that the anti-fibrotic effects of estrogen are not mediated by GPER.

Consistent with our earlier study, salt-loading in the estrogen-intact mRen2.Lewis rat resulted in early diastolic dysfunction [19]. The increasing degree of concentric remodeling with HS was associated with a decreased time required for deceleration of the early diastolic flow (E_dec_ time), which is indicative of reduced LV compliance [51]. Additionally, HS reduced early diastolic myocardial velocity, or e′, but did not increase LV filling pressure, as estimated by the E/e′ ratio. Although salt-induced cardiac hypertrophy and reduced systolic function adversely impacted LV lusitropy, endogenous estrogen most likely blunted the effect [19].

Along with its anti-remodeling action, the GPER agonist enhanced myocardial relaxation. Late administration of G-1 increased early mitral annular descent (e′) in NS and HS mRen2.Lewis rats. While reductions in vascular stiffening and lessening of hypertension can enhance diastolic function, systolic blood pressures were not overtly reduced by G-1 in this study. The increased e′ to a′ ratio further corroborates the direct lusitropic action of G-1 in the HS females. Importantly, an e′ to a′ ratio >1.0 is considered normal, with a reduced ratio (as in the HS vehicle group) indicating impaired early diastolic relaxation [52], [53]. Whether improvements in intracellular calcium handling and/or increased expression of calcium regulatory proteins partly explain the G-1-mediated lusitropic benefit remains to be investigated. Indeed, the trend toward enhanced systolic function in G-1 treated rats, particularly those fed NS, could have had positive influence on diastolic function. Two recent studies have shown that GPER activation improves contractile function and reduces infarct size in isolated hearts subjected to ischemia/reperfusion injury [14], [54].

The lack of an obvious effect of G-1 on blood pressure in the present study can be attributed to the presence of the endogenous ligand for GPER, estradiol. Although we did not assess circulating 17-β estradiol in the current study, we previously reported that serum estrogen levels are not changed with chronic salt loading [18]. Recently, we showed that chronic G-1 administration in ovariectomized mRen2.Lewis females with exacerbated hypertension significantly lowers systolic blood pressure to estrogen-intact levels but does not alter blood pressure in estrogen-intact littermates [15]. In addition, while the cardiac structural and functional effects of G-1 may also have been blunted by the presence of endogenous estradiol, the HS-induced increase in cardiac GPER expression may have allowed activation of the receptor in estrogen-intact rats.

A few limitations can be identified in this study. Although we found statistically significant differences in functional and structural outcomes, particularly early mitral annular descent and relative wall thickness, respectively, one potential concern is that our study may have been underpowered to reliably detect the effects of G-1 on blood pressure. Limited pilot data for a priori power analysis using G-1 under high salt conditions existed. Nevertheless, to place the differences in context, we observed a 28% increase in systolic blood pressure between salt conditions. Whereas, the G-1 differences in blood pressure ranged between −8% to 6% over the treatment period. These differences are unlikely to be clinically relevant even with a larger sample. Certainly, noninvasive blood pressure monitoring is less sensitive than radiotelemetry. However, this methodology was first validated in the early 1970s [55]. Since then, tail-cuff plethysmography has been reliably been used for conscious recordings in well-trained rats in our laboratory and that of others for the past 30 years, and it is well-accepted in hypertension research. Finally, the dose and duration of treatment of G-1 was based on our studies in the ovariectomized mRen2.Lewis rat [15]. Whether a prolonged duration or higher dose of G-1 could reduce blood pressure in this estrogen-intact, HS-fed model is not known. Dose-response studies are currently underway. Additionally, the structural effects of G-1 were only detected in the myocytes following this two-week regimen. We do not know beyond this time period, what effects there may be on fibrosis and collagen content, which may also change with a longer treatment period.

Taken together, these data provide the first evidence for a cardioprotective role for GPER in the hypertensive, salt-sensitive female mRen2.Lewis rat. Activation of this novel estrogen receptor attenuated diastolic dysfunction and cardiac hypertrophy in hypertensive female rats without requisite reductions in blood pressure. Given the lack of adequate therapeutic strategies for the treatment of diastolic dysfunction in females, targeting of GPER may elicit estrogenic benefits in the cardiovascular system. Clinically, GPER activation may offer a new hormone replacement approach providing less adverse effects than traditional therapy targeting all estrogen receptors.

## References

[1] Kitzman DW, Gardin JM, Gottdiener JS, Arnold A, Boineau R (2001). Importance of heart failure with preserved systolic function in patients > or  = 65 years of age. CHS Research Group. Cardiovascular Health Study.. Am J Cardiol.

[2] Masoudi FA, Havranek EP, Smith G, Fish RH, Steiner JF (2003). Gender, age, and heart failure with preserved left ventricular systolic function.. J Am Coll Cardiol.

[3] Solomon SD, Verma A, Desai A, Hassanein A, Izzo J (2010). Effect of intensive versus standard blood pressure lowering on diastolic function in patients with uncontrolled hypertension and diastolic dysfunction.. Hypertension.

[4] Weinberger MH (1991). Salt sensitivity as a predictor of hypertension.. Am J Hypertens.

[5] Heimann JC, Drumond S, Alves AT, Barbato AJ, Dichtchekenian V (1991). Left ventricular hypertrophy is more marked in salt-sensitive than in salt-resistant hypertensive patients.. J Cardiovasc Pharmacol.

[6] Garavaglia GE, Messerli FH, Schmieder RE, Nunez BD (1989). Sex differences in cardiac adaptation to essential hypertension.. Eur Heart J.

[7] Dubey RK, Jackson EK (2001). Cardiovascular protective effects of 17beta-estradiol metabolites.. J Appl Physiol.

[8] Pelzer T, Shamim A, Wölfges S, Schumann M, Neyses L (1997). Modulation of cardiac hypertrophy by estrogens.. Adv Exp Med Biol.

[9] Grohé C, Kahlert S, Löbbert K, Stimpel M, Karas RH (1997). Cardiac myocytes and fibroblasts contain functional estrogen receptors.. FEBS Lett.

[10] Filardo E, Quinn J, Pang Y, Graeber C, Shaw S (2007). Activation of the novel estrogen receptor G protein-coupled receptor 30 (GPR30) at the plasma membrane.. Endocrinology.

[11] Revankar CM, Mitchell HD, Field AS, Burai R, Corona C (2007). Synthetic estrogen derivatives demonstrate the functionality of intracellular GPR30.. ACS Chem Biol.

[12] Prossnitz ER, Arterburn JB, Smith HO, Oprea TI, Sklar LA (2008). Estrogen signaling through the transmembrane G protein-coupled receptor GPR30.. Annu Rev Physiol.

[13] Revankar CM, Cimino DF, Sklar LA, Arterburn JB, Prossnitz ER (2005). A transmembrane intracellular estrogen receptor mediates rapid cell signaling.. Science.

[14] Deschamps AM, Murphy E (2009). Activation of a novel estrogen receptor, GPER, is cardioprotective in male and female rats.. Am J Physiol Heart Circ Physiol.

[15] Lindsey SH, Cohen JA, Brosnihan KB, Gallagher PE, Chappell MC (2009). Chronic treatment with the G protein-coupled receptor 30 agonist G-1 decreases blood pressure in ovariectomized mRen2.Lewis rats.. Endocrinology.

[16] Chappell MC, Gallagher PE, Averill DB, Ferrario CM, Brosnihan KB (2003). Estrogen or the AT1 antagonist olmesartan reverses the development of profound hypertension in the congenic mRen2.Lewis rat.. Hypertension.

[17] Chappell MC, Westwood BM, Yamaleyeva LM (2008). Differential effects of sex steroids in young and aged female mRen2.Lewis rats: a model of estrogen and salt-sensitive hypertension.. Gend Med.

[18] Chappell MC, Yamaleyeva LM, Westwood BM (2006). Estrogen and salt sensitivity in the female mRen(2). Lewis rat.. Am J Physiol Regul Integr Comp Physiol.

[19] Groban L, Yamaleyeva LM, Westwood BM, Houle TT, Lin M (2008). Progressive diastolic dysfunction in the female mRen(2). Lewis rat: influence of salt and ovarian hormones.. J Gerontol A Biol Sci Med Sci.

[20] Whitesall SE, Hoff JB, Vollmer AP, D'Alecy LG (2004). Comparison of simultaneous measurement of mouse systolic arterial blood pressure by radiotelemetry and tail-cuff methods.. Am J Physiol Heart Circ Physiol.

[21] Junqueira LC, Bignolas G, Brentani RR (1979). Picrosirius staining plus polarization microscopy, a specific method for collagen detection in tissue sections.. Histochem J.

[22] Noorlander ML, Melis P, Jonker A, Van Noorden CJ (2002). A quantitative method to determine the orientation of collagen fibers in the dermis.. J Histochem Cytochem.

[23] Appleton CP (1991). Influence of incremental changes in heart rate on mitral flow velocity: assessment in lightly sedated, conscious dogs.. J Am Coll Cardiol.

[24] Choong CY, Herrmann HC, Weyman AE, Fifer MA (1987). Preload dependence of Doppler-derived indexes of left ventricular diastolic function in humans.. J Am Coll Cardiol.

[25] Smith SA, Stoner JE, Russell AE, Sheppard JM, Aylward PE (1989). Transmitral velocities measured by pulsed Doppler in healthy volunteers: effects of acute changes in blood pressure and heart rate.. Br Heart J.

[26] Stoddard MF, Pearson AC, Kern MJ, Ratcliff J, Mrosek DG (1989). Influence of alteration in preload on the pattern of left ventricular diastolic filling as assessed by Doppler echocardiography in humans.. Circulation.

[27] Dumesnil JG, Paulin C, Pibarot P, Coulombe D, Arsenault M (2002). Mitral annulus velocities by Doppler tissue imaging: practical implications with regard to preload alterations, sample position, and normal values.. J Am Soc Echocardiogr.

[28] Ommen SR, Nishimura RA, Appleton CP, Miller FA, Oh JK (2000). Clinical utility of Doppler echocardiography and tissue Doppler imaging in the estimation of left ventricular filling pressures: A comparative simultaneous Doppler-catheterization study.. Circulation.

[29] Nagueh SF, Kopelen HA, Quiñones MA (1996). Assessment of left ventricular filling pressures by Doppler in the presence of atrial fibrillation.. Circulation.

[30] Nagueh SF, Mikati I, Kopelen HA, Middleton KJ, Quiñones MA (1998). Doppler estimation of left ventricular filling pressure in sinus tachycardia. A new application of tissue doppler imaging.. Circulation.

[31] Bologa CG, Revankar CM, Young SM, Edwards BS, Arterburn JB (2006). Virtual and biomolecular screening converge on a selective agonist for GPR30.. Nat Chem Biol.

[32] Cleland JG, Swedberg K, Follath F, Komajda M, Cohen-Solal A (2003). The EuroHeart Failure survey programme—a survey on the quality of care among patients with heart failure in Europe. Part 1: patient characteristics and diagnosis.. Eur Heart J.

[33] Devereux RB, Roman MJ, Liu JE, Welty TK, Lee ET (2000). Congestive heart failure despite normal left ventricular systolic function in a population-based sample: the Strong Heart Study.. Am J Cardiol.

[34] Thohan V, Patel S (2009). The challenges associated with current clinical trials for diastolic heart failure.. Curr Opin Cardiol.

[35] Leenen FH, Yuan B (1998). Dietary-sodium-induced cardiac remodeling in spontaneously hypertensive rat versus Wistar-Kyoto rat.. J Hypertens.

[36] Lombardi WL, Gilbert EM (2000). The effects of neurohormonal antagonism on pathologic left ventricular remodeling in heart failure.. Curr Cardiol Rep.

[37] Filardo EJ, Thomas P (2005). GPR30: a seven-transmembrane-spanning estrogen receptor that triggers EGF release.. Trends Endocrinol Metab.

[38] Silvestre JS, Robert V, Heymes C, Aupetit-Faisant B, Mouas C (1998). Myocardial production of aldosterone and corticosterone in the rat. Physiological regulation.. J Biol Chem.

[39] Holycross BJ, Summers BM, Dunn RB, McCune SA (1997). Plasma renin activity in heart failure-prone SHHF/Mcc-facp rats.. Am J Physiol.

[40] Schunkert H, Danser AH, Hense HW, Derkx FH, Kürzinger S (1997). Effects of estrogen replacement therapy on the renin-angiotensin system in postmenopausal women.. Circulation.

[41] Sharkey LC, Holycross BJ, Park S, Shiry LJ, Hoepf TM (1999). Effect of ovariectomy and estrogen replacement on cardiovascular disease in heart failure-prone SHHF/Mcc- fa cp rats.. J Mol Cell Cardiol.

[42] de Jager T, Pelzer T, Müller-Botz S, Imam A, Muck J (2001). Mechanisms of estrogen receptor action in the myocardium. Rapid gene activation via the ERK1/2 pathway and serum response elements.. J Biol Chem.

[43] Brosnihan KB, Weddle D, Anthony MS, Heise C, Li P (1997). Effects of chronic hormone replacement on the renin-angiotensin system in cynomolgus monkeys.. J Hypertens.

[44] Nickenig G, Bäumer AT, Grohè C, Kahlert S, Strehlow K (1998). Estrogen modulates AT1 receptor gene expression in vitro and in vivo.. Circulation.

[45] Roesch DM, Tian Y, Zheng W, Shi M, Verbalis JG (2000). Estradiol attenuates angiotensin-induced aldosterone secretion in ovariectomized rats.. Endocrinology.

[46] Haugen E, Chen J, Wikström J, Grönros J, Gan LM (2007). Parallel gene expressions of IL-6 and BNP during cardiac hypertrophy complicated with diastolic dysfunction in spontaneously hypertensive rats.. Int J Cardiol.

[47] Nakagawa O, Ogawa Y, Itoh H, Suga S, Komatsu Y (1995). Rapid transcriptional activation and early mRNA turnover of brain natriuretic peptide in cardiocyte hypertrophy. Evidence for brain natriuretic peptide as an “emergency” cardiac hormone against ventricular overload.. J Clin Invest.

[48] Yang CM, Kandaswamy V, Young D, Sen S (1997). Changes in collagen phenotypes during progression and regression of cardiac hypertrophy.. Cardiovasc Res.

[49] Xu Y, Arenas IA, Armstrong SJ, Davidge ST (2003). Estrogen modulation of left ventricular remodeling in the aged heart.. Cardiovasc Res.

[50] Dai Q, Lin J, Craig T, Chou YM, Hinojosa-Laborde C (2008). Estrogen effects on MMP-13 and MMP-14 regulation of left ventricular mass in Dahl salt-induced hypertension.. Gend Med.

[51] Oh JK, Hatle L, Tajik AJ, Little WC (2006). Diastolic heart failure can be diagnosed by comprehensive two-dimensional and Doppler echocardiography.. J Am Coll Cardiol.

[52] Dumesnil JG, Paulin C, Pibarot P, Coulombe D, Arsenault M (2002). Mitral annulus velocities by Doppler tissue imaging: practical implications with regard to preload alterations, sample position, and normal values.. J Am Soc Echocardiogr.

[53] Nagueh SF, Sun H, Kopelen HA, Middleton KJ, Khoury DS (2001). Hemodynamic determinants of the mitral annulus diastolic velocities by tissue Doppler.. J Am Coll Cardiol.

[54] Bopassa JC, Eghbali M, Toro L, Stefani E (2010). A novel estrogen receptor GPER inhibits mitochondria permeability transition pore opening and protects the heart against ischemia-reperfusion injury.. Am J Physiol Heart Circ Physiol.

[55] Buñag RD (1973). Validation in awake rats of a tail-cuff method for measuring systolic pressure.. J Appl Physiol.

